# TCF7L2 polymorphisms, nut consumption, and the risk of metabolic syndrome: a prospective population based study

**DOI:** 10.1186/s12986-021-00542-7

**Published:** 2021-01-12

**Authors:** Somayeh Hosseinpour-Niazi, Bahar Bakhshi, Asiyeh-Sadat Zahedi, Mahdi Akbarzadeh, Maryam S. Daneshpour, Parvin Mirmiran, Fereidoun Azizi

**Affiliations:** 1 Nutrition and Endocrine Research Center, Research Institute for Endocrine Sciences, Shahid Beheshti University of Medical Sciences, Tehran, Iran; 2 Cellular and Molecular Research Center, Research Institute for Endocrine Sciences, Shahid Beheshti University of Medical Sciences, No 24, A’rabi St, Yeman Av, P.O. Box 19395-4763, Velenjak, Tehran Iran; 3 Department of Clinical Nutrition and Dietetics, Faculty of Nutrition Sciences and Food Technology, National Nutrition and Food Technology Research Institute, Shahid Beheshti University of Medical Sciences, No 24, A’rabi St, Yeman Av, P.O. Box 19395-4763, Velenjak, Tehran Iran; 4 Endocrine Research Center, Research Institute for Endocrine Sciences, Shahid Beheshti University of Medical Sciences, Tehran, Iran

**Keywords:** Nuts, Metabolic syndrome, TCF7L2 polymorphisms, Gene-diet interaction, Weight change

## Abstract

**Background:**

The aim of this study was to investigate whether two variants of the TCF7L2 (rs7903146 and rs12255372) modify the association between nut consumption and the risk of metabolic syndrome (MetS). Additionally, the modifying effect of weight change during follow-up on these associations was investigated.

**Material and methods:**

We prospectively studied 1423 participants of the Tehran Lipid and Glucose study aged 19–74 years who were followed-up for dietary assessment using a validated, semi-quantitative food frequency questionnaire. Multivariable-adjusted Cox regression was used to estimate hazard ratios (HRs) for MetS events. Genotyping was performed by Human Omni Express-24-v1-0 chip.

**Results:**

Over a median 8.9 years of follow-up, 415 new cases of MetS were documented. The median nut consumption was 20.0 g/week (Interquartile Range (IQR): 8.6–38.9 g/week). Regarding the rs7903146 genotype, in carriers of T allele (CT + TT), highest tertile of nut consumption was associated with a reduced risk of MetS after adjusting for confounders (HR: 0.67 (0.50–0.91)). Regarding the rs12255372 genotype, highest versus lowest tertile of nut consumption in participants with T allele (GT + TT) resulted in 34% reduction of MetS risk after adjustment for confounders (HR: 0.66 (0.49–0.69)). After stratification by weigh change (< 7% or ≥ 7% weight gain), in individuals with ≥ 7% weight gain, highest tertile of nut consumption was associated with reduced risk of MetS among the risk allele of rs7903146. In the risk allele of rs12255372, among individuals with < 7% weight gain, third tertile of nuts intake reduced the risk of MetS, after adjustment for confounders.

**Conclusion:**

Higher consumption of nuts may reduces the risk of MetS in T-risk allele of the TCF7L2 rs7903146 and rs12255372 variants and weight change may modify this association.

## Introduction

Metabolic syndrome (MetS) is a multicomponent condition characterized by insulin resistance, dyslipidemia, abdominal obesity, and hypertension that has well-documented associations with an increased risk of type 2 diabetes mellitus, cardiovascular disease (CVD), and atherosclerosis [1, 2]. MetS has a multifactorial etiology, involving both genetics and modifiable environmental factors, including dietary determinants [3]. Identifying potential genes that are associated with MetS and investigating the modifying effect of genetics on the association of dietary determinants with the risk of MetS are among novel approaches for preventing and treating MetS. This approach is based on the precision-based healthcare that enables use of therapeutic diets and dietary recommendations according to patients’ genetic background that eventually, will lead to better prevention and treatment of MetS [4].

Among different single nucleotide polymorphisms (SNPs), the transcription factor-7-like 2 (TCF7L2) rs7903146 and rs12255372 are assumed to be associated with type 2 diabetes as well as MetS in different populations [5, 6]. For the first time, the Diabetes Prevention Program and the Diabetes Prevention Study reported that lifestyle intervention reduces diabetic risk among individuals with genetic susceptibility of TCF7L2 risk genotypes [7, 8]. Followed by this investigation, current literature suggests associations of this gene with the risk of type 2 diabetes, obesity, and MetS while considering the modulating effects of some dietary determinants, such as whole-grains [9], glycemic index and glycemic load [10], dietary fiber [11], low fat and high carbohydrate diet [12], dietary saturated fatty acids (SFA) [13], western dietary pattern [14], and prudent dietary pattern [15]. However, to the best of our knowledge, the interaction between nut consumption and polymorphisms of this gene has not yet been investigated. Nuts, as rich sources of fiber, polyphenols, mono- and polyunsaturated fatty acids, vitamins, minerals, and protein [16], could potentially reduce the risk of chronic diseases [17–21]. Although the inverse association between nut consumption and BMI, overweight, and obesity are documented in previous research [22, 23], limited studies have assessed the interaction between obesity, nut consumption, and chronic disease [24, 25]. Moreover, recently, an interaction between TCF7L2 and obesity in relation to type 2 diabetes has been reported [26, 27]. Therefore, the aim of the current study was to investigate (1) the association of nut consumption with the MetS risk based on the TCF7L2 rs7903146 and rs12255372 genotypes, and (2) whether these associations are modified according to weight change during follow-up (≥ or < 7%) among adult participants of this population-based study, over a median 8.9-year of follow-up.

## Materials and methods

### Study population

We conducted a prospective population-based study within the framework of the Tehran Lipid and Glucose Study (TLGS). The TLGS is an ongoing prospective study, aiming to prevent non-communicable diseases through promoting a healthy lifestyle. The detailed TLGS method, described elsewhere [28], was initiated in March 1999. Multistage, stratified cluster random sampling technique was used for enrollment of > 15,000 individuals aged ≥ 3 years from Tehran’s urban district 13, a district with a population representative of the urban population of Tehran, the capital city of Iran. Beginning in 1999 with 3-year intervals afterwards, participants were assessed for demographic and lifestyle factors, socioeconomic status, medication use, medical history of cardiovascular risk factors, and anthropometric measures. To update all the previous data, the information were documented every 3 years during face-to-face visits by the local research team. Phases II, III, IV, V, VI were prospective follow-up studies conducted between 2002–2004, 2006–2007, 2008–2011, 2012–2015, 2016–2018, respectively. The current study used the baseline examination data from phase III of the TLGS (2006–2007) and followed participants up to phase VI of TLGS (2016–2018), during an 8.91-year follow-up (Interquartile range (IQR): 7.98–9.69).

**Fig. 1:**
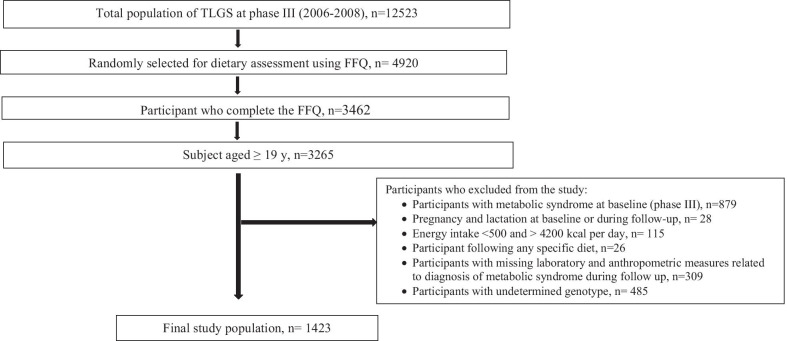
Flowchart of the study population, Tehran Lipid and Glucose Study (2006–2008 to 2016–2018)

During the third examination survey of the TLGS (2006–2007), a number of 12,523 participants were assessed for medical history and physical examinations. Subsequently, a representative sample of 4920 participants, based on their age and gender, was randomly selected to complete the dietary assessment. From the 4920 selected participants in the present study, 3462 number of individuals, with similar characteristics to the total population in phase III of the TLGS, agreed to complete the food frequency questionnaire (FFQ) [29]. For the current study, of 3462 participants, 3265 adults aged 19–74 years with complete demographic, anthropometric, biochemical, and dietary data were selected. Moreover, participants with MetS at baseline (n = 879), participants who were pregnant or lactating at baseline and during follow-up (n = 28), participants with daily energy intake < 500 and > 4200 kal per day (n = 115), participants following any specific diet (n = 26), and participants with missing laboratory and anthropometric measures related to diagnosis of MetS during follow up (n = 309) and participants with undetermined genotype (n = 485) were excluded from the study. The final analysis was conducted based on the data from 1423 participants, from 2006 until 2018, during the 8.9-year follow-up (Fig. 1).

This study protocol has been approved by the ethics committee of the Research Institute for Endocrine Sciences (RIES), Shahid Beheshti University of Medical Sciences (No. IR.SBMU.ENDOCRINE.REC.1398.084), and written informed consent was obtained from all participants.

### Dietary assessment

A validated, semi-quantitative food frequency questionnaire (FFQ) was used to obtain and update dietary intake information every 3 years [30]. The primary FFQ had 168 food items in 2006 then the food items were decreased to 147 in 2009, due to the low frequency of consumption of certain food items and combining selected food items. Consumption frequencies of each food item on a daily, weekly, or monthly basis during the previous year were documented according to a standard unit or portion size, evaluated in household measures specified for each food, during face-to-face interviews by expert dietitians, and consumed portion sizes were converted to grams. Since the Iranian food composition table (FCT) is incomplete, the US Department of Agriculture (USDA) FCT (https://ndb.nal.usda.gov/ndb/ accessed 15 March 2019) was used to calculate energy, macro- and micronutrients. Total energy, macro- and micronutrient content were calculated by summing up consumption of these nutrients. Due to the crucial impact of recent dietary intakes on the association of diet and chronic disease, in the present study we used an alternative approach, providing more weight to the recent diet, to reduce within subject variations [31]. From the initial number of 1423 participants at baseline, 453 participants completed all 4 FFQs, 499 participants completed 3 FFQs, 278 participants completed 2 FFQs, and 193 participants did not complete the any FFQs during follow-up. To impute missing values, last observation carried forward method was used [31].

Nut consumption collected using the FFQ showed a valid estimate against multiple dietary records. A good correlation coefficient was observed between FFQ and multiple 24 recalls (0.54 and 0.39 in males and females, respectively) and between two FFQs (0.34 and 0.52 in males and females, respectively) [32]. Moreover, there was reasonable reliability, validity and stability of the dietary patterns, using data from the FFQ, over the 8-year follow-up period [33].

### Biochemical assessment

At baseline and follow-up, between 7∶00 and 9∶00 a.m., venous blood samples were obtained from all study participants who fasted for 12–14 h over the previous night. The blood samples were placed into vacutainer tubes, and were centrifuged within 30–45 min of collection. Fasting blood glucose (FPG), high density lipoprotein (HDL-C), and triglyceride (TG) analysis were done at the TLGS research laboratory on the day of sample collection, using a selector 2 auto-analyzer (Vital Scientific, Spankeren, the Netherlands) and commercial kits (Pars Azmoon Inc., Tehran, Iran). FPG was analyzed using an enzymatic colorimetric method with the glucose oxidase technique; inter- and intra-assay coefficients of variation (CV) at baseline and follow-up phases were both less than < 2.3%. TG was assayed using an enzymatic colorimetric method with glycerol phosphate oxidase. HDL-C was analyzed after precipitation of apolipoprotein B-containing lipoproteins with phosphotungstic acid. Both intra- and inter-assay CVs were below 2.1 and 3.0% for TG and HDL-C, respectively, in all baseline and follow-up assays. All samples were analyzed when internal quality control met the acceptable criteria.

### Assessment of other variables

At the baseline recruitment, data on characteristics of participants were collected by trained researchers using a standardized questionnaire, which included demographic information, lifestyle factors (smoking status and physical activity), socioeconomic status (education, and employment), medication regimen (antihypertensive, lipid-lowering and anti-diabetic agents), past medical history. Details of physical assessment including weight, height, waist circumference, and blood pressure have been documented elsewhere [28]. Briefly, participants’ weight, while being minimally clothed without wearing shoes, was measured using a digital scale (Seca 707: range 0–150 kg) and recorded with accuracy of 100 g. Height was measured in a standing position, with shoulders in normal alignment, without shoes, using Seca 225 stadiometer (Seca GmbH) and recorded to the nearest 0.5 cm. BMI was calculated by dividing weight in kilograms into height per square meter. Waist circumference (WC) was measured at the umbilical level using an un-stretched tape measure, without any pressure to the body surface, and was recorded to the accuracy of 0.5 cm. After participants rested in a sitting position for 15 min, blood pressure was measured using a standardized mercury sphygmomanometer (calibrated by the Iranian Institute of Standards and Industrial Researches) on the right arm twice at least 30 s apart, and the average of the two measurements was reported as the blood pressure. Physical activity was assessed using modifiable activity questionnaire (MAQ) that included a list all three forms of activities, including leisure time, job, and household activities; the frequency and amount of time spent per week engaged in physical activity over the last year were recorded [34]. Physical activity levels were expressed as metabolic equivalent hours per week (METsh/week). The reliability and convergent validity of the Persian version of the MAQ had been reported [35].

### Definition of metabolic syndrome

In accordance with the Joint Interim Statement, diagnosis of MetS required the presence of three or more of the following criteria [1]: (1) elevated glucose concentrations (FPG concentration ≥ 100 mg/dL or treatment with anti-hyperglycemic medications), (2) TG concentration (≥ 150 mg/dL or treatment with anti-hypertriglyceridemia medications), (3) reduced serum HDL-C (< 50 mg/dL in women and < 40 mg/dL in men), (4) elevated blood pressure (≥ 130/85 mmHg or treatment with anti-hypertensive medications), and (5) enlarged abdominal circumference (≥ 95 cm according to the population- and country-specific cut-off points for Iranian adults of both genders) [36].

### Genotyping

Genomic DNA was extracted from the buffy-coat of samples using a proteinase K/salting out standard method [37]. The quality of extracted DNA was determined using the Nanodrop 1000 Spectrophotometer. Samples in the range of 1.7 < A260/A280 < 2 were excluded due to low quality and concentration.

DNA samples were processed on HumanOmniExpress-24-v1-0 bead chips (containing 649,932 SNP loci with an average mean distance of 4 kb) at the deCODE genetics company (Reykjavik, Iceland) according to the manufacturer’s specifications (Illumina Inc., San Diego, CA, USA). Quality control procedures performed by PLINK program (V 1.07) and R statistic (V 3.2) [38]. Then the genotyping data of two TCF7L2 polymorphisms (rs7903146 and rs12255372) was used for association analysis. In our study population, the TCF7L2 SNPs (rs12255372 and rs7903146) showed moderate linkage disequilibrium (r^2^ = 0.67).

### Statistical analysis

Statistical analyses were performed using SPSS package, version 17.0 (SPSS, Chicago, IL). All tests were two-tailed, and P values < 0.05 were considered as statistically significant. The significance of deviations of observed genotype frequencies from those predicted by the Hardy–Weinberg equation were evaluated with χ2 test. Based on TCF7L2 rs7903146 and rs12255372 genotypes, participants were categorized according to major allele homozygotes, heterozygotes, and minor allele homozygotes (additive model). Chi-square for categorical data and general linear models for continuous data were used to compared baseline characteristics and dietary variables across tertiles of nut consumption. Hazard ratio (95% confidence interval) was computed using Cox proportional hazards regression analyses. The first model was crude. The second model was adjusted for age, gender, smoking, physical activity, education levels, occupational status, total energy intake, fiber intake, family history of diabetes, family history of cardiovascular disease. Model 3 was further adjusted for BMI at baseline. Regarding the interaction between weight change during follow-up and rs7903146 and rs12255372 genotypes (three categories) on the risk of MetS, we stratified participants according to their weight change (< 7% and ≥ 7%) and investigated the effect modification of weight change on the association between nut consumption and risk of MetS based on rs7903146 and rs12255372 genotypes.

## Results

Genotype distributions were in Hardy–Weinberg equilibrium (P = 0.50 for rs12255372 and P = 0.27 for rs7903146). Considering the rs7903146 genotypes, the frequency of CT and TT were 46.5% and 14.7%, respectively, whereas the frequency of GT and TT genotypes were 44.9% and 13.0% in rs12255372. Genotype effect on MetS risk found for both rs7903146 and rs12255372 genotypes after adjustment for age, gender and BMI (Table 1). Participants with the CT and TT genotypes of the rs7903146 had a HR of 1.21 (0.97–1.49) and 1.47 (1.10–1.95) for incident MetS, compared with CC genotype, whereas HR (95% CIs) of MetS were 1.21 (0.98–1.49) and 1.47 (1.09–1.98) for those with the rs12255372 GT and TT genotypes, respectively.

**Table 1:** Genotypes and allele frequency of TCF7L2 SNPs

	CC		CT		TT		C allele frequency		T allele frequency	
	Non-MetS	MetS	Non-MetS	MetS	Non-MetS	MetS	Non-MetS	MetS	Non-MetS	MetS
Rs7903146										
Total population	407	145	461	200	140	70	1275	490	741	340
Weigh change < 7%	234	58	309	92	93	18	777	208	495	128
Weight change ≥ 7%	173	87	152	108	47	52	498	282	246	212

	GG		GT		TT		G allele frequency		T allele frequency	
	Non-MetS	MetS	Non-MetS	MetS	Non-MetS	MetS	Non-MetS	MetS	Non-MetS	MetS
Rs12255372										
Total population	440	159	443	196	125	60	1323	514	693	316
Weigh change < 7%	255	67	296	82	85	19	806	216	466	120
Weight change ≥ 7%	185	92	147	114	40	41	517	298	227	196

Number of participants

We documented 415 new cases of MetS. The median nut consumption was 20.0 g/week (IQR: 8.6–38.9 g/week). Baseline characteristics of the study participants based on both rs7903146 genotype and tertiles of nut consumption are presented in Table 2. There was no significant difference in age, gender, physical activity, occupation status, family history of diabetes, family history of CVD, and BMI across tertiles of nut consumption in rs7903146 SNP. Participants in the highest tertile of nuts intake who were carries of rs7903146 T allele were less likely to be smokers and those with CC genotype was more educated. Participants in highest tertile of nut consumption were more likely to have higher energy intakes, dietary fiber, fruit, and vegetable intakes. Additionally, participants with CC genotype who were in the highest tertile of nuts intake consumed more dairy products, poultry, fish, Meat, processed meat and organ meat and legume. Participants with CT genotype consumed more dairy products and dietary cholesterol and participants with TT genotype consumed more dietary cholesterol, whole grain, refined grain, legume and dairy products.

**Table 2:** Baseline characteristics and intake of dietary variables of study population across tertiles of nut consumption according to transcription factor-7-like 2 rs7903146 genotype

rs7903146 genotype	CC		P value	CT		P value	TT		P value
	T1	T3		T1	T3		T1	T3	
Age (y)	35.2 ± 0.9	37.7 ± 0.9	0.16	37.6 ± 0.8	37.9 ± 0.8	0.62	35.0 ± 1.6	35.7 ± 1.6	0.82
Female (%)	61.5	55.7	0.41	40.7	36.0	0.49	53.5	65.7	0.33
Physical activity (MET h-week)	4.3 ± 0.3	4.2 ± 0.3	0.45	4.3 ± 0.3	4.4 ± 0.3	0.58	4.2 ± 0.3	4.3 ± 0.3	0.51
Current smoking (%)	21.5	19.6	0.63	22.6	15.1	0.04	29.6	14.3	0.05
Academic degrees (%)	19.5	33.0	0.01	20.4	24.9	0.14	26.8	22.9	0.48
Occupational status, employed (%)	41.0	45.6	0.46	40.4	43.4	0.72	50.7	32.9	0.08
Family history of diabetes (%)	33.3	37.6	0.76	35.4	28.9	0.62	32.4	28.6	0.08
Family history of CVD events (%)	16.9	16.0	0.73	38.9	40.9	0.98	25.4	10.0	0.08
BMI at baseline (kg/m^2^)	25.7 ± 0.3	25.9 ± 0.3	0.89	25.7 ± 0.3	26.0 ± 0.3	0.11	25.7 ± 0.5	25.0 ± 0.5	0.64
*Dietary variables*									
Total energy (Kcal/d)	2123 ± 87	2562 ± 88	0.002	2106 ± 44	2460 ± 44	< 0.001	2181 ± 81	2640 ± 81	< 0.001
Carbohydrate (% of total energy)	60.3 ± 1.0	61.8 ± 1.0	0.17	61.2 ± 0.7	61.7 ± 0.7	0.82	059.5 ± 1.2	61.5 ± 1.2	0.43
Protein (% of total energy)	14.3 ± 0.9	14.5 ± 0.9	0.23	14.5 ± 0.2	14.6 ± 0.2	0.19	14.0 ± 0.3	14.5 ± 0.3	0.06
Fat (% of total energy)	30.8 ± 0.4	30.5 ± 0.4	0.13	29.6 ± 0.3	30.0 ± 0.3	0.69	31.0 ± 0.6	30.4 ± 0.6	0.75
SFA (% of total energy)	10.3 ± 0.2	9.7 ± 0.2	0.11	9.9 ± 0.2	9.6 ± 0.2	0.44	9.7 ± 0.3	9.6 ± 0.3	0.22
MUFA (% of total energy)	10.7 ± 0.3	10.2 ± 0.3	0.47	10.1 ± 0.1	10.1 ± 0.1	0.98	10.8 ± 0.3	10.1 ± 0.3	0.13
PUFA (% of total energy)	6.2 ± 0.1	6.3 ± 0.1	0.32	5.9 ± 0.1	6.2 ± 0.1	0.14	6.6 ± 0.2	6.2 ± 0.2	0.11
Total fiber (g/d)	37.9 ± 1.2	47.2 ± 1.2	< 0.001	39.2 ± 1.2	46.1 ± 1.2	< 0.001	39.6 ± 2.2	49.8 ± 2.2	< 0.001
Cholesterol (g/d)	200 ± 24	258 ± 24	0.07	209 ± 7.5	240 ± 7.6	0.018	202 ± 12	255 ± 12	0.013
Vegetable (g/d)	247 ± 11	338 ± 11	< 0.001	247 ± 9	320 ± 9	< 0.001	246 ± 15	333 ± 15	< 0.001
Fruit (g/d)	279 ± 18	497 ± 18	< 0.001	303 ± 20	472 ± 20	< 0.001	270 ± 32	510 ± 32	< 0.001
Meat, processed meat and organ meat(g/d)	23.9 ± 0.3	33.3 ± 1.3	< 0.001	24.8 ± 1.4	28.4 ± 1.4	0.12	24.3 ± 2.5	31.7 ± 2.5	0.09
Poultry and fish (g/d)	33.0 ± 2.9	46.8 ± 2.9	< 0.001	35.5 ± 6	150 ± 6	0.06	32.3 ± 3.8	44.5 ± 3.9	0.06
Whole grain (g/d)	137 ± 6	151 ± 7	0.37	139 ± 6	150 ± 6	0.25	115 ± 9.8	158 ± 9.9	0.01
Refined grain (g/day)	336 ± 12	315 ± 12	0.43	322 ± 9	314 ± 9	0.31	378 ± 19	342 ± 19	0.04
Legumes (g/d)	31.0 ± 1.8	39.8 ± 1.8	0.001	35.9 ± 1.6	39.6 ± 1.6	0.10	35.3 ± 3.9	50.1 ± 3.9	0.007
Dairy products (g/d)	353 ± 16	420 ± 16	0.013	344 ± 14	410 ± 14	0.004	336 ± 25	435 ± 26	0.02

Data are express as mean ± SE, unless otherwise indicated

Dietary data were adjusted for kcal

Table 3 presents the multivariable-adjusted HR for the MetS risk across tertiles of nut consumption according to TCF7L2 rs7903146 and rs12255372 genotype, in total population. Regarding the rs7903146 variant, in participants with CT genotype, highest tertile of nut consumption was associated with a 36% reduction in the risk of MetS in crude model (HR: 0.64, CI (0.45–0.90)), this association remained significant after further adjustments in model 2 and 3 (HR: 0.61, CI (0.43–0.87) and HR: 0.63, CI (0.44–0.90), respectively). Additionally, carriers of T-allele (CT + TT) in the third tertile of nut consumption had a significantly lower risk of MetS in all 3 statistical models, a 33% reduction of risk in crude model, a 34% reduction of risk in model 2 and a 33% reduction in model 3 (HR: 0.67, CI (0.50–0.90), HR: 0.66, CI (0.49–0.89), and HR: 0.67, CI (0.50–0.91), respectively). As for the rs12255372 variant, highest versus lowest tertile of nut consumption in participants with GT genotype resulted in a 32% and 33% reduction of MetS risk in model 2 and model 3 (HR: 0.68, CI (0.48–0.96) and HR: 0.67, CI (0.47–0.95), respectively). In individuals with TT genotype, third tertile of nut consumption was associated with a lower risk of MetS in model 1, this association diminished to non-significant levels after further adjustments. Moreover, in carriers of T-allele (CT + TT) consumption of nuts significantly reduced the risk of MetS in all 3 models.

**Table 3:** Hazard ratios of metabolic syndrome across tertiles of nut consumption according to transcription factor-7-like 2 rs7903146 and rs12255372 genotypes

	Tertiles of nut consumption			
	T1	T2	T3	P_trend_^a^
*Rs7903146*				
CC				
Model 1	1	0.95 (0.63–1.42)	0.97 (0.65–1.45)	0.97
Model 2	1	0.88 (0.58–1.33)	0.84 (0.55–1.27)	0.71
Model 3	1	0.85 (0.56–1.29)	0.82 (0.54–1.25)	0.63
CT				
Model 1	1	0.71 (0.51–0.99)	0.64 (0.45–0.90)	0.02
Model 2	1	0.67 (0.47–0.94)	0.61 (0.43–0.87)	0.01
Model 3	1	0.70 (0.49–0.99)	0.63 (0.44–0.90)	0.02
TT				
Model 1	1	0.80 (0.46–1.39)	0.61 (0.34–1.11)	0.27
Model 2	1	0.79 (0.44–1.41)	0.64 (0.35–1.18)	0.36
Model 3	1	0.78 (0.43–1.42)	0.72 (0.39–1.34)	0.56
CT + TT				
Model 1	1	0.73 (0.55–0.97)	0.67 (0.50–0.90)	0.01
Model 2	1	0.70 (0.52–0.93)	0.66 (0.49–0.89)	0.01
Model 3	1	0.72 (0.54–0.97)	0.67 (0.50–0.91)	0.02
*Rs12255372*				
GG				
Model 1	1	0.94 (0.64–1.38)	1.04 (0.71–1.52)	0.86
Model 2	1	0.83 (0.55–1.24)	0.89 (0.60–1.32)	0.65
Model 3	1	0.80 (0.53–1.19)	0.92 (0.62–1.37)	0.54
GT				
Model 1	1	0.78 (0.56–1.10)	0.77 (0.55–1.08)	0.18
Model 2	1	0.74 (0.52–1.04)	0.68 (0.48–0.96)	0.04
Model 3	1	0.75 (0.53–1.07)	0.67 (0.47–0.95)	0.03
TT				
Model 1	1	0.53 (0.29–0.98)	0.49 (0.26–0.91)	0.03
Model 2	1	0.58 (0.31–1.09)	0.63 (0.32–1.26)	0.18
Model 3	1	0.66 (0.34–1.26)	0.70 (0.35–1.39)	0.39
GT + TT				
Model 1	1	0.69 (0.51–0.93)	0.69 (0.51–0.93)	0.03
Model 2	1	0.68 (0.51–0.92)	0.66 (0.49–0.90)	0.02
Model 3	1	0.69 (0.51–0.93)	0.66 (0.49–0.69)	0.01

Model 1 was crude

Model 2 was adjusted for age, gender, smoking, physical activity, education levels, occupational status, total energy intake, fiber intake, family history of diabetes, family history of cardiovascular disease

Model 3 was additionally adjusted for BMI at baseline

^a^The median intake of each tertile category was assigned and then these quartile median variables were included as a continuous variable in cox proportional hazards regression

An interaction of MetS risk was found for nut consumption, TCF7L2 variants, and weigh change during follow-up. In participants with < 7% weight gain during the follow-up, the multivariable-adjusted HRs (95% CI) for the MetS risk across tertiles of nut consumption according to both TCF7L2 rs7903146 and rs12255372 genotypes are presented in Table 4. In the rs12255372 GT genotype, third tertile of nuts intake reduced the risk of MetS by 47% in model 2 (HR: 0.53, CI (0.30–0.94). At last, GT + TT individuals in the highest tertile of nut consumption had a 46% reduction MetS risk in model 2 (HR: 0.54, CI (0.32–0.90). No association was found between nut consumption and risk of MetS among rs7903146 genotypes. Table 5 presents the multivariable-adjusted HRs (95% CI) for the MetS risk across tertiles of nut consumption according to both TCF7L2 rs7903146 and rs12255372 genotype among participant with a weight gain ≥ 7%. Regarding rs7903146, in subjects with CT genotype, nut consumption was associated with a lower risk of MetS after adjustment for cofounding factors **(**highest vs. lowest tertile of consumption: HR: 0.49, CI (0.30–0.79)). CT + TT individuals in the highest tertile of nut consumption had a 45% reduction MetS risk after adjustment for confounding factors **(**HR: 0.55, CI (0.37–0.82). No association was found between nut consumption and risk of MetS among rs12255372 genotypes.

**Table 4:** Hazard ratios of metabolic syndrome across tertiles of nut consumption according to transcription factor-7-like 2 rs7903146 and rs12255372 genotype among participant with weigh change < 7% during follow-up

	Tertiles of nut consumption			
	T1	T2	T3	P_trend_^a^
*Rs7903146*				
CC				
Model 1	1	0.80 (0.42–1.50)	0.86 (0.46–1.62)	0.78
Model 2	1	0.76 (0.39–1.46)	0.69 (0.35–1.36)	0.53
CT				
Model 1	1	0.76 (0.48–1.31)	0.85 (0.52–1.39)	0.73
Model 2	1	0.81 (0.49–1.34)	0.72 (0.42–1.21)	0.32
TT				
Model 1	1	0.69 (0.24–1.99)	0.46 (0.13–1.54)	0.44
Model 2	1	0.51 (0.16–1.61)	0.35 (0.09–1.36)	0.27
CT + TT				
Model 1	1	0.73 (0.46–1.14)	0.75 (0.48–1.18)	0.30
Model 2	1	0.74 (0.46–1.17)	0.63 (0.39–1.02)	0.13
*Rs12255372*				
GG				
Model 1	1	0.66 (0.36–1.25)	1.07 (0.61–1.88)	0.29
Model 2	1	0.70 (0.36–1.35)	0.89 (0.47–1.65)	0.40
GT				
Model 1	1	0.92 (0.55–1.54)	0.70 (0.41–1.21)	0.19
Model 2	1	0.85 (0.50–1.43)	0.53 (0.30–0.94)	0.02
TT				
Model 1	1	0.58 (0.21–1.65)	0.40 (0.12–1.30)	0.15
Model 2	1	0.63 (0.21–1.89)	0.95 (0.21–4.20)	0.68
GT + TT				
Model 1	1	0.81 (0.51–1.29)	0.66 (0.41–1.07)	0.11
Model 2	1	0.76 (0.48–1.22)	0.54 (0.32–0.90)	0.02

Model 1 was crude

Model 2 was adjusted for age, gender, smoking, physical activity, education levels, occupational status, total energy intake, fiber intake, family history of diabetes, family history of cardiovascular disease

^a^The median intake of each tertile category was assigned and then these quartile median variables were included as a continuous variable in cox proportional hazards regression

**Table 5:** Hazard ratios of metabolic syndrome across tertiles of nut consumption according to transcription factor-7-like 2 rs7903146 and rs12255372 genotype among participant with weigh change ≥ 7% during follow-up

	Tertiles of nut consumption			
	T1	T2	T3	P_trend_^a^
*Rs7903146*				
CC				
Model 1	1	1.21 (0.72–2.03)	1.05 (0.62–1.78)	0.74
Model 2	1	1.33 (0.77–2.26)	105 (0.61–1.81)	0.51
CT				
Model 1	1	0.58 (0.37–0.91)	0.54 (0.33–0.85)	0.01
Model 2	1	0.57 (0.35–0.92)	0.49 (0.30–0.79)	0.007
TT				
Model 1	1	1.14 (0.59–2.20)	0.91 (0.45–1.83)	0.80
Model 2	1	1.25 (0.58–2.69)	0.69 (0.31–1.51)	0.27
CT + TT				
Model 1	1	0.70 (0.48–1.01)	0.59 (0.40–0.87)	0.02
Model 2	1	0.67 (0.46–0.97)	0.55 (0.37–0.82)	0.009
*Rs12255372*				
GG				
Model 1	1	1.02 (0.62–1.71)	1.04 (0.63–1.72)	0.98
Model 2	1	1.06 (0.63–1.80)	1.09 (0.65–1.82)	0.94
GT				
Model 1	1	0.58 (0.36–1.12)	0.75 (0.49–1.16)	0.37
Model 2	1	0.57 (0.35–1.12)	0.74 (0.47–1.17)	0.38
TT				
Model 1	1	0.85 (0.41–1.77)	0.68 (0.31–1.46)	0.34
Model 2	1	0.60 (0.25–1.43)	0.36 (0.14–1.10)	0.14
GT + TT				
Model 1	1	0.60 (0.41–1.09)	0.71 (0.49–1.03)	0.16
Model 2	1	0.60 (0.45–1.09)	0.71 (0.49–1.03)	0.10

Model 1 was crude

Model 2 was adjusted for age, gender, smoking, physical activity, education levels, occupational status, total energy intake, fiber intake, family history of diabetes, family history of cardiovascular disease

^a^The median intake of each tertile category was assigned and then these quartile median variables were included as a continuous variable in cox proportional hazards regression

## Discussion

In this population-based prospective study, the protective effect of higher nut consumption against the MetS incidence depended on individuals’ genetic background. In our total population, higher nut consumption reduced risk of MetS among carriers of a single T-risk allele of the TCF7L2 rs7903146 and rs12255372 variants. In rs7903146 variant, CT + TT individuals with high weight gain experienced a lower risk of MetS; however, considering rs12255372 genotype, higher nut consumption reduced risk of MetS only among participants with low weight gain.

With respect to gene-diet associations, previous prospective cohort and interventional studies have reported modifying effects of the TCF7L2 polymorphisms on the diet-disease associations [9, 11–13, 39–42]. In line with our findings suggesting risk-lowering effects of nut consumption on MetS in participants with genotypes CT + TT rs7903146 and GT + TT rs12255372 risk, data have reported a protective effect of other dietary compounds, as well as dietary patterns, against type 2 diabetes risk in subjects carrying the risk-allele T of the TCF7L2 variants [39–41]. For instance, in a PREDIMED randomized controlled trial [40], adherence to the Mediterranean diet resulted in lower fasting plasma glucose and lipid profile concentrations among TCF7L2 rs7903146 TT genotype. Moreover, after a median 4.8 years of follow-up, the Mediterranean diet attenuated the risk of stroke in TT individuals who were at a higher genetic susceptibility to stroke [40]. In addition, regarding dietary compounds with detrimental effects on health, a prospective study has documented that dietary fatty acids’ type and amount of consumption could potentially exacerbate the risk of developing MetS in individuals with high genetic susceptibility, as reported in the study, high consumption of SFAs and low consumption of polyunsaturated fatty acids (PUFAs) increased the MetS risk in rs7903146 T allele carriers [13]. On the contrary, some of the existing evidence has suggested the protective effects of dietary compounds, such as dietary fiber and cereals, on type 2 diabetes risk among individuals with C homozygote genotype of the TCF7L2. As demonstrated by the EPIC-Potsdam case-cohort study, the inverse association of whole-grain intake and type 2 diabetes risk was only present in the rs7903146 C homozygote individuals, namely, the T-allele carriers could not benefit from the risk-reducing effects of whole-grains [9]. Similarly, a cross-sectional analysis of the Malmö Diet and Cancer Study Cardiovascular Sub-cohort, indicated that a higher fiber intake was associated with a reduced risk of type 2 diabetes and lower HbA1C levels in individuals with the CC genotype of the TCF7L2 rs7903146 variant; however, individuals carrying T-risk allele lacked this protective effect [11]. Furthermore, results from the Stockholm Diabetes Prevention Program resembled the above-mentioned findings, preventive effects of whole-grains and cereals against developing type 2 diabetes in individuals with the non-risk C allele, with no observed associations in carriers of the T risk-allele [42].

BMI, as a main confounder, modulates the association of nut consumption and hazard of chronic disease [25]. In the current study, long-term weight gain of participants modified the effects of nut consumption on the MetS risk, based on rs12255372 and rs7903146 polymorphisms of the TCF7L2 gene, suggesting an interplay of BMI and genetic susceptibility on the association of nut consumption and MetS incidence. The effect of TCF7L2 variants on risk of type 2 diabetes and obesity as well as modulation effect of BMI on the association between diabetes and TCF7L2 variants, have been controversial [26, 27, 43]. This discrepancy, at least in part, may be attributed to the extent of linkage disequilibrium in different population [8, 44]. In the Diabetes Prevention Program, both rs12255373 and rs7903146 predicted progression to diabetes [7], whereas in the Finnish Diabetes Prevention Study only rs12255372 was significantly associated with the risk of diabetes [8]. In European population, TCF7L2 has not been a risk factor for obesity, but obesity modulates the association between TCf7l2 and risk of type 2 diabetes, additionally, the rs7903146 T allele was more associated with T2D in non-obese individuals than in obese subjects [45].

Moreover, findings regarding the interaction between dietary determinants and TCF7L2 variants (rs12255372 and rs7903146) on anthropometric measures are controversial. In Tuebingen Lifestyle Intervention Program (TULIP), reduction in BMI and adiposity were observed for both common genotypes of rs12255372 and rs7903146 [46]. In addition, Mediterranean diet significantly interacted with 2 SNPs (rs122255372 and rs7903146) on anthropometric measures; higher Mediterranean score associated with lower BMI and weight among risk allele carriers participants [41]. In contrast, in a randomized control trial (RCT), consumption of low fat diet in overweight individuals with risk allele of rs12255372 reduced BMI and fat mass; however, no interaction was found between TCF7L2 rs7903146 and dietary intervention on BMI and adiposity [47]. In another RCT, consumption of wheat bread and nopal tortilla diet, high in dietary fiber, decreased BMI in both CC and CT/TT genotypes of the TCF7L2 rs7903146 variant, yet no significant effect of rs12255372-T risk allele on anthropometric and metabolic indicators was observed [48]. In addition, an interaction between TCF7L2 SNP rs12255372, but not rs7903146, and fiber intake (g/day) on HDL-C was reported [49].

In the current study, we found that nut consumption have greater risk reducing effects on MetS among ≥ 7 weight gain group with genotype rs7903146, while this risk reduction was obvious among participants with < 7 weight gain group with rs12255372 genotype. In addition, this effect was only observed among participants with heterozygote T genotypes. Lack of observed association between nut consumption and risk of MetS among homozygote T carriers may be due to lower sample size in this population, thereby having insufficient power to detect an association; a major problem that was shown in genetic associating studies [50]. In the current study statistical power for detection of risk of MetS among TT genotypes of the TCF7L2 rs7903146 and rs12255372 variants in total population (0.145–0.464) as well as based on weight changes (0.143–0.756) during follow-up was low; suggesting that due to low power, it is not possible to get a definitive result. Therefore our finding needed to be repeated in other ethnic population with different allele frequency, family-based investigations as well as studies with large sample size.

Some strengths and limitations of this study should be acknowledged. First and foremost, its population-based prospective design, use of a validated of FFQ, a long enough follow-up, and the availability of data on potential confounders provided strength for this study. However, dietary assessment using FFQ may lead to measurement error, which eventually, places individuals into wrong categorize of intake [51]. As declared before, due to fewer number of individuals with TT genotype, there is possibility that our study lacked the sufficient power to detect an association of nut consumption and MetS risk in this population. Further research with higher sample size with regard to the TCF7L2 risk genotypes is suggested.

## Conclusions

Higher consumption of nuts may reduce the risk of MetS in T-risk allele of the TCF7L2 rs7903146 and rs12255372 variants and weight change may modify this association.

## Data Availability

The datasets generated and/or analysed during the current study are not publicly available due institution’s policy but are available from the corresponding author on reasonable request.

## Abbreviations

MetS: Metabolic syndrome
CVD: Cardiovascular disease
SNPs: Single nucleotide polymorphisms
TCF7L2: Transcription factor-7-like 2
SFA: Saturated fatty acids
TLGS: Tehran lipid and glucose study
IQR: Interquartile range
FFQ: Food frequency questionnaire
RIES: Research Institute for Endocrine Sciences
USDA: US Department of Agriculture
CV: Coefficients of variation
FPG: Fasting blood glucose
HDL-C: High density lipoprotein
TG: Triglyceride
WC: Waist circumference
METsh/week: Metabolic equivalent hours per week
PUFAs: Polyunsaturated fatty acids
